# Myxoid liposarcoma: local relapse and metastatic pattern in 43 patients

**DOI:** 10.1186/s12885-018-4226-8

**Published:** 2018-03-20

**Authors:** Hans Roland Dürr, Jessica Rauh, Andrea Baur-Melnyk, Thomas Knösel, Lars Lindner, Falk Roeder, Volkmar Jansson, Alexander Klein

**Affiliations:** 1 Musculoskeletal Oncology, Department of Orthopaedic Surgery, Physical Medicine and Rehabilitation, Campus Grosshadern, University Hospital, LMU Munich, Marchioninistr. 15, 81377 Munich, Germany; 2 Institute of Radiology, Campus Grosshadern, University Hospital, LMU Munich, Marchioninistr. 15, 81377 Munich, Germany; 3 Institute of Institute of Pathology, Campus Grosshadern, University Hospital, LMU Munich, Marchioninistr. 15, 81377 Munich, Germany; 4 Department of Medicine III, Campus Grosshadern, University Hospital, LMU Munich, Marchioninistr. 15, 81377 Munich, Germany; 5 Department of Radiation Oncology, Campus Grosshadern, University Hospital, LMU Munich, Marchioninistr. 15, 81377 Munich, Germany; 6 CCU Radiation Oncology, German Cancer Research Center (DKFZ), Heidelberg, Germany

**Keywords:** Sarcoma, Myxoid liposarcoma, Surgery, Recurrence, Prognosis, Survival

## Abstract

**Background:**

Liposarcomas are the second most common type of soft tissue sarcomas, 30–50% of these are of myxoid subtype. The aim of this retrospective study was to analyze the local control rate, the metastatic pattern and survival of patients in a consecutive single-institution series.

**Methods:**

From 1983 to 2015, 43 patients with myxoid liposarcoma of the extremities and trunk wall underwent resections. The margin was defined as R0 (wide) or R1 (marginal). Patients were followed for evidence of local recurrence or distant metastasis. Overall and recurrence-free survival was calculated.

**Results:**

The mean age was 48.6 years. The lower extremity was involved in 40 cases, the mean tumour size was 12 cm. In 31 cases a wide and in 12 cases a marginal resection was performed. Grading was G1 in 14, G2 in 25 and G3 in 4 cases.

Nine patient died in follow-up, 4 of them with metastatic disease, all nonpulmonary. 5-year local recurrence (LR) free survival was 82%. 4 (9.3%) patients developed LR (all R1). Overall survival (OS) was 81% after 5 and 72% after 10 years. In multivariate analysis age and Grading proved to be significant on OS. According to univariate analysis, only age over 48 years and distant metastasis had a significant impact on overall survival.

**Conclusions:**

Patients with myxoid liposarcomas have a good prognosis. Myxoid liposarcoma has a distinct pattern of nonpulmonary metastatic disease. Therefore, patients with high-risk extremity myxoid liposarcoma should undergo imaging studies of the chest, abdomen, spine and pelvis as part of their staging and follow-up examinations preferably with whole body MRI, or CT scans and MRI of the spine and pelvic region for detection of suspected metastatic disease.

## Background

Sarcomas in adult patients comprise approximately 1% of all newly diagnosed cancers with an incidence of 3–6 cases per 100,000 population [1, 2]. Liposarcomas are the second most common type of soft tissue sarcomas (15–20%), 30–50% of these are of myxoid or myxoid round cell subtype [3–5]. Myxoid liposarcoma is a genetically distinct variant of liposarcoma, characterized by a t(12:16) translocation. A variable content of round cells is characteristic and known as a poor prognostic factor (> 5%) [5, 6].

From a clinical point of view an important factor of this usually slow-growing, deep seated tumour mainly located in the lower extremities is the propensity to metastasize to nonpulmonary soft-tissues as the retroperitoneum, the bone or the contralateral limb [7]. Furthermore, myxoid liposarcoma is particular radiosensitive thus neoadjuvant radiation protocols may be very effective [8–10].

The aim of this retrospective study was to analyze the local control rates, the metastatic pattern and survival of patients in a consecutive single-institution series.

## Methods

From 1983 to 2015, 43 consecutive patients with myxoid liposarcoma of the extremities and trunk wall had been treated in our institution, 37 of them since 2005. All tumours were located deep to the fascia and had a diagnosis of myxoid liposarcoma based on histological features and immunohistochemistry. Thirty-seven patients had primary, 6 recurrent disease.

Preoperative staging included at least MRI (predominantly) or CT of the primary tumour region and CT chest. All patients underwent limb-sparing surgical resection. The margin was defined as R0 if a rim of sound tissue around the lesion was present (wide resection) or R1 if the margins were contaminated but the tumour capsule closed (marginal resection). The French Federation of Cancer Centers grading system was used to assign tumour grade [11]. All patients were followed routinely for evidence of local recurrence or distant metastasis.

For statistical analysis, overall and recurrence-free survival were calculated by the Kaplan-Meier method. Univariate subgroup analysis were done using the log-rank test (time-to-event data) or the chi-square test. For multivariate analysis a Cox proportional-hazard regression model was used. Significance analysis was performed using the log-rank test, the Chi-Square test or the Cox proportional-hazards regression. The data analysis software used was MedCalc®.

## Results

The mean age of the 21 male and 22 female patients was 48.6 years (range, 18–83). The lower extremity was involved in 40 cases (25 thigh, 9 lower calf, 5 popliteal region, 1 ft), the upper arm in 1 and the trunk in 2 patients. The mean tumour size was 12 cm (range, 1–33).

The mean duration of symptoms was 15 months (range, 0–71), 35 (81%) patients complained of swelling, 5 (12%) of pain. Neurological impairment (sensory) or restriction of movement was seen occasionally. Forty patients had a biopsy or histology from previous surgeries, in 3 cases surgery was done as excisional biopsy. In 3 patients metastatic disease was evident at the time of surgery (2 primary, one of them also in local recurrence). The metastatic lesions were found in the retroperitoneal space, the lumbar spine and the lymph nodes of the pelvis.

In 31 cases a wide (R0) resection and in 12 a marginal (R1) resection was achieved. In wide resections the margins were smaller than 1 mm in 9 patients, between 1 and 5 mm in 11 and larger than 5 mm in 11 patients. Grading was G1 in 14, G2 in 25 and G3 in 4 cases.

Surgical complications included transient neurapraxia in 7 patients, prolonged wound healing in 10, hematoma or seroma in 7, lymphedema in 3, infection in 1 and fractures in 3 (all after periostal resection and radiotherapy). In total 14 surgical revisions had to be performed. Twenty-one patients received postoperative and 11 patients preoperative radiation therapy. Indication for radiation therapy was seen generally in patients with G2/G3 lesions or after marginal resection in G1 lesions. Chemotherapy of variable protocols had been applied in 22 patients.

Nine patient died in follow-up, 4 of them had proven metastatic disease disease. The median follow-up of the surviving was 46 months (range, 2–305). Nine patients had a follow-up of less than 24 months, 4 of them less than 12 months.

**Fig. 1:**
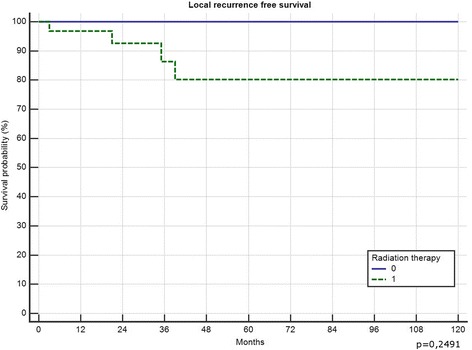
Effect of radiotherapy on local recurrence free survival

**Fig. 2:**
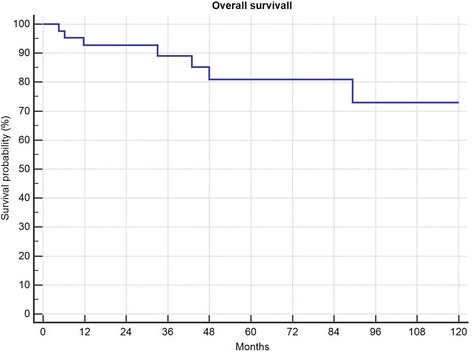
Overall survival in 43 patients with myxoid liposarcoma

**Fig. 3:**
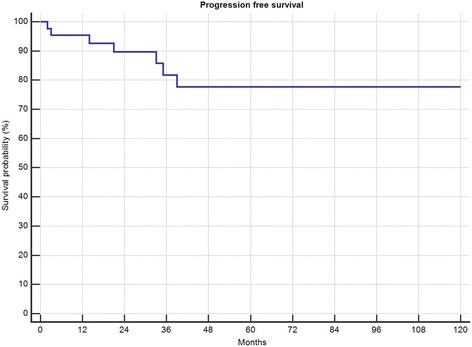
Progression free survival in 43 patients with myxoid liposarcoma

**Fig. 4:**
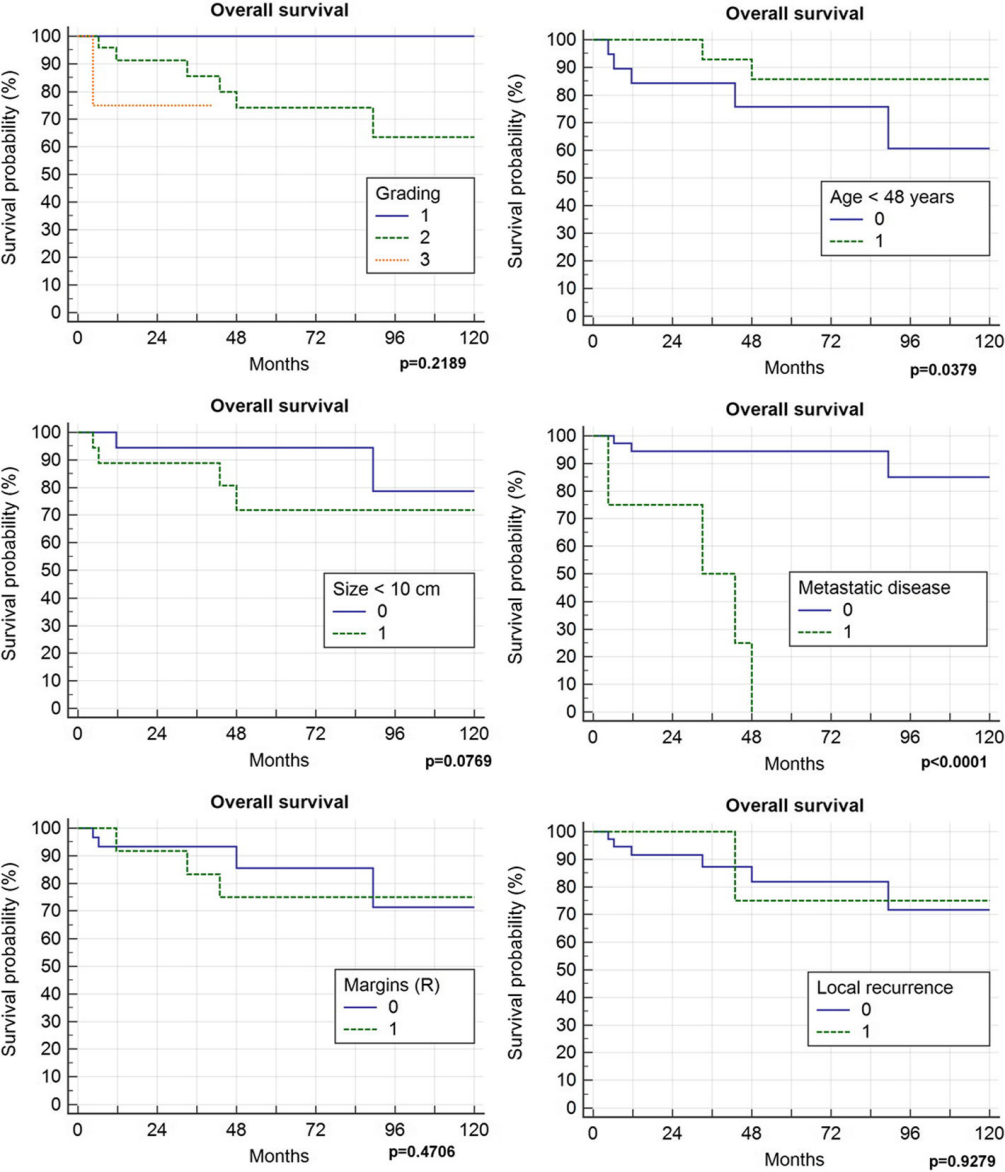
Overall survival in dependence to different clinical parameters

5-year local recurrence free survival was 82%. In total 4 (9.3%) patients developed local recurrences 21, 34, 42 and 49 months after surgery. All of them had a marginal (R1) resection (4/12 R1, 0 of 31 R0; *p* = 0.0034) and suffered from G2 tumours (n.s.). Two of them had primary tumours, 2 had been treated for locally recurrent disease (*p* = 0.034). All 4 received radiation therapy (2 preoperative, 2 postoperative). Only one of those 4 had metastatic disease (initially) and deceased in follow-up. Local recurrence free survival in those patients with risk factors necessitating radiation therapy was not significantly different than the local recurrence free survival in patients without (Fig. 1). In one patient metastatic disease developed 2 months after resection of a primary tumour retro- and intraperitoneal, all 3 patients with preexisting metastatic disease showed progression. Overall survival was 81% after 5 years and 72% after 10 years (Fig. 2), progression free survival was 78% after 5-years (Fig. 3). Patients with primary tumours had a mean overall survival of 196 months in recurrent disease 60 months respectively (n.s.). In multivariate analysis the patient and tumour-dependent factors as age, Grading and size proved to be significant on overall survival. Including recurrent disease, only age and grading remained significant (Table 1). According to univariate analysis, only age over 48 years and presence or development of distant metastasis had a significant impact on overall survival (Fig. 4).

**Table 1:** Cox proportional-hazards regression of grading and size of the tumor and age of the patient

Factor	*P*	HR	95% CI
Age	0,0027	1,16	1,05–1,27
Grading	0,0239	26,30	1,54–448,91
Size	0,1961	1,07	0,97–1,18
Primary/Recurrent	0,9607	0,00	0

HR indicates hazards ratio; CI 95% confidence interval

## Discussion

The crude rate of local recurrences (LR) and distant metastasis (DM) was 9% each. Overall survival (OS) was 81% after 5 years and 72% after 10 years. These results seem to compare favourably with published series reporting LR rates of 13–33%, DM rates of 11–38% ad 10-year OS rates of 55–86%, although age distribution and tumour sizes (our mean 12 cm, published 9–14 cm) were similar in our compared to the mentioned studies [12–18]. The in literature described shorter OS in recurrent cases failed significance in this series. We identified tumour size, grading and age with a cut off of 45–60 years as prognostic factors regarding overall survival according to multivariate analysis [13, 15, 18, 19]. In contrast, we could not verify a significant negative impact of recurrent disease with regard to OS as reported by other investigators, probably due to the small number of patients. Obviousely these patients are a selected group as they have already recurred. They are likely to be more biologically aggressive as evidenced by the fact that out of our 6 patients 3 (50%) developed a relapse (2 local and 1 distant).

As shown in Fig. 4 grading has an impact, mainly between grade 1 and 2, weaker between 2 and 3, as also described by Fiore et al. [17]. We did not compare the amount of the round cell component with a cut-off level of 5% to differentiate pure myxoid liposarcoma (MLPS) from round cell liposarcoma (RCLPS). MLPS tumours always were graded 1, there as RCLPS may reflect the worse survival in in the group of G2 or 3 tumours.

Metastatic disease has a strong impact on survival with a poor outcome. All our 4 patients who had MD at diagnosis or developed MD in the course of the disease died with a median survival of 33 months. This comparatively long survival in MD stands in contrast with other metastasized soft tissue sarcomas but represents the experience of other authors, too [7, 20]. The distinctive pattern of MD is well known in myxoid liposarcoma. Common sites are the retroperitoneum, abdominal wall and abdominal cavity [7]. Schwab et al. reported 17% of patients developing skeletal metastases, more than one-half of metastatic events in their series [21]. Estourgie et al. described 13 of 14 patients with MD had extrapulmonary lesions [20]. Because of MD developing also in soft tissues, multicentric lesions at diagnosis should be seen critically and may represent in fact MD [22–24]. This metastasis in fat-bearing areas is unique and might be contributed by the expression of high levels of adipophilin and chemokine (C-X-C motif) receptor 4 (CXCR4), which are both correlated with adipogenesis and metastasis [25].

Conventional methods of imaging as chest x-rays, CT or even PET-scans well established in other soft tissue sarcomas may fail in detecting metastastic disease in myxoid liposarcoma.

**Fig. 5:**
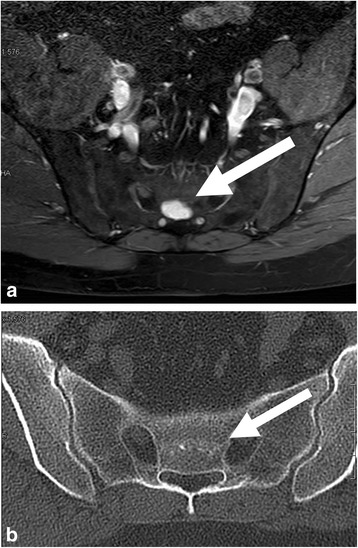
**a**-**c** 41-year old patient with solitary metastatic disease in the sacrum (S3) 6 years after surgery of the primary in the right calf. Lesion clearly detectable on MRI (**a**) but not on CT (**b**) or PET-CT scan (**c**)

In one of our metastatic patients, even FDG-PET-CT was not able to detect the skeletal lesion as shown in Fig. 5 [26–28]. In a 2010 published survey all 8 patients with skeletal MD were positive on MRI, 2 out of 4 were negative in bone scans and 6 out of 8 were negative in CT scans [29]. Therefore WBMRI seems to be the most reliable method at present. For example Seo et al. demonstrated sensitivity and specifity rates of 80%/97% in soft-tissue lesions and 85%/99% in bony lesions respectively in a series of 15 patients [30]. Stevenson et al. reported 28 patients who received whole-body MRI and CT. Of 38 lesions found on MRI, 29 were located inside a corresponding CT field of view but only 5 of 8 soft-tissue lesions and none of 21 bony lesions were detected [31].

Patients with positive surgical margins had a 30% risk of LR compared to 0% in wide resections. But this did not influence OS neither if we correlated it to margins nor to LR itself. Only one out of 4 patients with LR developed DM. In literature this is discussed controversially. Margins seem to influence disease specific survival but not metastasis-free survival [32]. This may be caused by the inclusion of patients with critical locations as retroperitoneum or head and neck in this studies, not included in this series. Further on, we found a clear but insignificant trend between larger tumour size and the presence of contaminated margins.

Interestingly, in this study radiation therapy had a great influence on therapy by decreasing the size of many tumours in a significant proportion, whereas it did not influence LR. This is in contrast to Guadagnolo et al. from the MDACC where in 11 patients with positive surgical margins and radiotherapy (median dose 50 Gy) no one showed LR [8]. Chung et al. from Toronto published a 98% LR-free 5-year survival with only 2 recurrences in 88 patients [9]. Using radiotherapy in marginal or even intralesional resections in 10 patients the addition of radiotherapy resulted in no local relapse at a mean follow-up of 58 months [33]. Even in a hypofractionated protocol (5 × 5 Gy followed by immediate surgery) in 32 patients a 5-year LR of 10% was achieved [34]. In many other retrospective studies, as in ours, in which radiation is part of therapy in selected cases a strong bias with more relevant risk factors in patients in the radiotherapy group might influence the result. However, the effect of neoadjuvant radiotherapy on tumour size could be seen in many of our cases. This was not part of this study protocol but as described by Pitson et al. in 16 patients 50 Gy of preoperative radiotherapy induced a significant reduction of 59% in the mean MRI tumour volume [10].

Chemotherapy has been applied in many of our cases due to a general regime in soft tissue sarcomas in our sarcoma center. Going back in literature doxorubicin- and dacarbazine-based chemotherapy had been proven to be effective in myxoid liposarcoma [35]. More recently trabectedin had shown beneficial effects both as second-line chemotherapy and in neoadjuvant protocols [36, 37].

## Conclusion

In summary patients with myxoid liposarcomas generally have a good prognosis. Overall survival was 72% after 10 years, local recurrence was seen only in 9% of the patients treated with limb-sparing surgery and risk-adapted radiation therapy. Preoperative radiation therapy further provides a substantial effect in decreasing tumour size. This particular subtype of soft tissue sarcomas has specific characteristics as a distinct pattern of nonpulmonary metastatic disease. Therefore, patients with high-risk extremity myxoid liposarcoma should undergo imaging studies of the chest, abdomen, spine and pelvis as part of their staging and follow-up examinations preferably with whole body MRI, or CT scans and MRI of the spine and pelvic region for detection of suspected metastatic disease.

## Abbreviations

CI: Confidence interval
cm: Centimeter
CT: Computed tomography
DM: Distant metastasis
FDG-PET: Fluordesoxyglucose-positron emission tomography
G1, G2, G3: Grading according to the French Federation of Cancer Centers grading system
HR: Hazard ratio
LR: Local recurrence
MD: Metastatic disease
MLPS: Pure myxoid liposarcoma
MRI: Magnetic resonance imaging
n.s.: Not significant
OS: Overall survival
R0, R1, R2: Resection margin
RCLPS: Round cell liposarcoma
WBMRI: Whole-body magnetic resonance imaging
